# A Psychometric Tool for Evaluating Executive Functions in Parkinson’s Disease

**DOI:** 10.3390/jcm11051153

**Published:** 2022-02-22

**Authors:** Francesca Borgnis, Francesca Baglio, Elisa Pedroli, Federica Rossetto, Mario Meloni, Giuseppe Riva, Pietro Cipresso

**Affiliations:** 1 IRCCS Fondazione Don Carlo Gnocchi ONLUS, 20148 Milan, Italy; fborgnis@dongnocchi.it (F.B.); fbaglio@dongnocchi.it (F.B.); frossetto@dongnocchi.it (F.R.); mmeloni@dongnocchi.it (M.M.); 2 Department of Psychology, Università Cattolica del Sacro Cuore, 20123 Milan, Italy; giuseppe.riva@unicatt.it; 3 Applied Technology for Neuro-Psychology Lab, Istituto Auxologico Italiano, Istituto di Ricovero e Cura a Carattere Scientifico, 20149 Milan, Italy; e.pedroli@auxologico.it; 4 Faculty of Psychology, eCampus University, 22060 Novedrate, Italy; 5 Department of Psychology, Università degli Studi di Torino, 10124 Turin, Italy

**Keywords:** executive function, 360° environment, assessment, virtual reality, Parkinson’s disease, usability, user experience, engagement, patients

## Abstract

Recently, there has been an increasing interest in using 360° virtual-reality video for an ecologically valid assessment of executive functioning in the neurologic population. In this framework, we have developed the EXecutive-functions Innovative Tool (EXIT 360°), an original 360°-based instrument for a multicomponent, ecologically valid evaluation of executive functioning in Parkinson’s Disease (PD). This work aimed to test the usability and user experience of EXIT 360° in patients with PD (PwPD). Twenty-seven PwPD and twenty-seven healthy controls underwent an evaluation that involved: (1) usability assessment by the System Usability Scale and (2) evaluation of user experience using the ICT—Sense of Presence and User Experience Questionnaire. Results showed a satisfactory level of usability for patients (mean = 76.94 ± 9.18) and controls (mean = 80 ± 11.22), with good scores for usability and learnability. Regarding user experience, patients provided a positive overall impression of the tool, evaluating it as attractive, enjoyable, activating, and funny. Moreover, EXIT 360° showed good pragmatic (e.g., efficient, fast, clear) and hedonic quality (e.g., exciting, interesting, and creative). Finally, PwPD considered EXIT 360° as an original tool with high ecological validity (mean = 4.29 ± 0.61), spatial presence (mean = 3.11 ± 0.83) and engagement (mean = 3.43 ± 0.54) without relevant adverse effects. Technological expertise had no impact on performance. Overall, EXIT 360° appeared to be a usable, easy-to-learn, engaging, and innovative instrument for PD. Further studies will be conducted to deepen its efficacy in distinguishing between healthy subjects and patients with executive dysfunctions.

## 1. Introduction

Over the years, virtual reality-based (VR) tools have appeared to be a promising solution in neuropsychological assessment, providing an ecological evaluation to detect everyday cognitive impairments [1,2,3]. Specifically, several studies have shown the feasibility, acceptability, and efficacy of VR-based tools in the early assessment and rehabilitation of executive dysfunctions (ED) in many neurologic pathologies, for example, Parkinson’s disease (PD) [4,5,6,7].

ED constitutes a typical non-motor symptom in PD, from the early stages of the disease [8,9,10,11], with a negative impact on everyday functioning and quality of life [12,13,14,15]. Specifically, patients with PD showed several impairments in planning, attention, working memory, set-shifting, dual-task performance, inhibitory control, and decision making, also compromising social–cognition abilities [5,16,17]. Thus, patients have trouble in many essential goal-directed daily activities, with repercussions on everyday functioning (i.e., preparing meals, managing money, shopping, and work) [6,14,18,19]. Moreover, a growing number of longitudinal studies have suggested that early ED is predictive of subsequent development of PD dementia [11,20,21]. Therefore, the early identification of executive impairments could identify individuals with PD at risk of developing dementia, providing the opportunity for timely neurorehabilitation interventions [6,22]. In this framework, an early and ecologically valid evaluation of daily executive impairments appears crucial to achieving excellent disease management. Thus, VR-based instruments that allow for carrying out different everyday tasks in ecologically valid and controlled environments [23,24,25,26] appear to be a promising solution in the early evaluation of ED.

A preliminary study conducted using a virtual supermarket showed the presence of a deficit in planning and the switching mechanism required to process, in parallel, a large volume of information [27,28]. VR allowed for a description of planning alterations by testing “pure” mental sequences without the interference of possible motor deficits.

In the following years, the virtual version of the Multiple Errand Test (VMET) allowed for the detection of several executive disorders typical in patients with PD [6,29,30]. In a preliminary study, Raspelli and colleagues have confirmed the presence of deficits in planning and showed impairments in problem solving, set shifting, and sustained attention (few strategies and much perseveration) [29]. The following study conducted by Albani and collaborators also showed deficits in decision making in individuals with PD (more errors and fewer effective strategies than controls) [30]. Similar results were found by Cipresso and colleagues that showed the presence of impairments in cognitive flexibility in PD with normal cognition [6]. This study has shown that an evaluation in real-life context provides a more accurate estimate of the patient’s impairment, hidden in traditional measures: patients with PD differ from healthy controls subjects in VMET performance but not in the conventional neuropsychological assessment of EFs. Therefore, a more ecologically valid evaluation of executive functions (EFs) leads to better detection of subtle deficits since the early stage of PD. In recent years, some authors have exploited the advanced 360° technology in the evaluation of executive functioning in PD [25]. To date, 360° video appears to be a promising interactive virtual technology for creating virtual-reality–immersive applications at a low cost [31]. Implementing neuropsychological tests in 360° environments is an actual challenge; in this framework, Serino and colleagues have developed and validated a 360° version of a paper-and-pencil test for EFs known as the Picture Interpretation Test (PIT) [25]. Results showed the efficacy of PIT 360° as a highly sensitive ecological tool for detecting deficits in active visual perception from the early stages of PD. The traditional neuropsychological test for executive functioning did not differentiate between patients and healthy controls. 

In light of these interesting results, Borgnis and colleagues (2021) have developed a 360° instrument to allow for an ecologically valid and multicomponent evaluation of executive functioning: the EXecutive-functions Innovative Tool 360° (EXIT 360°) [32]. In EXIT 360°, participants are engaged in a “game for health” delivered via a standard mobile-powered VR headset, in which they are immersed in 360° domestic environments and have to perform seven everyday subtasks developed to evaluate many components of executive functioning simultaneously and quickly. After developing EXIT 360°, the same authors conducted two preliminary studies to assess the convergent validity of EXIT 360° and usability, involving healthy control subjects [33]. The first study showed a significant correlation between the EXIT 360° score and several standardized neuropsychological tests for EFs (Borgnis et al., submitted); therefore, EXIT 360° can be considered as a 360°-based tool able to assess several components of executive functioning (Borgnis et al., submitted). The second study showed promising and interesting results regarding usability, user experience, and engagement of EXIT 360° [33]. First, participants obtained a positive global opinion of the instrument, judging it as usable, easy-to-learn, clear, enjoyable, attractive, and friendly. Second, EXIT 360° appeared as an efficient and fast tool, with excellent hedonic quality in terms of stimulation (exciting and interesting) and originality. Moreover, EXIT 360° also seemed to be an engaging and challenging device with high spatial presence, excellent ecological validity, and irrelevant adverse sides. Finally, data on healthy control subjects showed that demographic characteristics and technological expertise had no impact on the performance.

Several studies have shown the need to consider the evaluation of “usability” and “user experience” as crucial elements in developing technological tools [34,35,36,37]. Sauer and colleagues recently introduced a new higher-level concept, “interaction experience,” that integrated these two critical aspects, providing major benefits to users and improving their experience with technological instruments [35]. The usability assessment allows for comprehending the “degree to which a subject can use a system to achieve specific goals effectively, efficiently, and satisfactorily within a well-defined context of use” [38]. Therefore, its evaluation allows for understanding of any technical difficulties affecting subjects’ performance. Moreover, cybersickness (e.g., nausea, vertigo, dizziness, headache, sweating) can result in unpleasant experiences, impacting the users’ performance and significantly decreasing the test results’ validity. For this reason, work on user experience appears crucial. Previous studies have underlined the importance of improving the user experience in virtual environments by working on five domains in digital content development: a sense of presence, a sense of realism, engagement, enjoyment, and side effects [39]. Focusing on enjoyment and attractiveness increases users’ motivation and participation and reduces the anxiety of neuropsychological evaluation. Finally, another critical aspect of evaluation regards the technological expertise, above all older adults, since poor performance could be due to insufficient technological expertise [40]. 

Despite the evident importance of paying attention to usability and user experience, only one study has been conducted to evaluate the usability of VR-based instruments for the assessment of neurocognitive abilities in Parkinson’s disease. Pedroli and colleagues conducted a preliminary study involving 21 healthy control subjects and three patients with PD in an evaluation with VMET. Data showed that healthy participants gave good usability for the VMET, while the patients with PD showed that there needs more than an improvement to VMET to be considered usable. Moreover, results showed that a good training phase before the test is crucial to apply the virtual protocol to PD patients [37].

This work aimed to test the usability and user experience of EXIT 360° in patients with PD. 

## 2. Materials and Methods

### 2.1. Participants

Twenty-seven patients with PD (PwPD) (M:F = 11:16) and 27 healthy control subjects (HC) (M:F = 11:16) matched for age and education were consecutively recruited at IRCCS Fondazione Don Carlo Gnocchi ONLUS in Milan. All participants had to follow specific inclusion criteria: (a) age between 18 and 90 years; (b) education ≥5 (primary school); (c) absence of cognitive impairment as determined by the Montreal Cognitive Assessmen*t* test [41] (MoCA score ≥17.54, the cut-off of normality), corrected for age and years of education according to Italian normative data [42]; and (d) ability to provide written, signed informed consent. Moreover, PwPD had to meet the following inclusion criteria: (a) clinically established or probable Parkinson’s disease according to Movement Disorder Society (MDS) criteria [43]; (b) mild to moderate disease staging, with scores <3 on the Hoehn and Yahr scale; and (c) deficits in EFs confirmed by documented neurological and/or neuropsychological evaluation. Exclusion criteria for all subjects were: (a) severe hearing or visual impairments, (b) major systemic, psychiatric, or other neurological illnesses and (c) overt visual hallucinations or vertigo.

The study was approved by the “Fondazione Don Carlo Gnocchi-Milan” Ethics Committee on 7 April 2021, project identification code 09_07/04/2021. The neuropsychologist provided all participants with a complete explanation of the purpose and risk of the study before they signed the written informed consent based on the revised Declaration of Helsinki [44]. 

### 2.2. Procedure

All participants underwent a one-session evaluation at IRCCS Fondazione Don Carlo Gnocchi ONLUS in Milan that involved three main phases: (a) pre-task evaluation; (b) EXIT 360° session; and (c) post-task evaluation [45].

In pre-task evaluation (a), all participants underwent an assessment of their global cognitive profile through the MoCA test, a sensitive screening tool able to exclude the presence of cognitive impairment [41,42]. Moreover, the psychology evaluated their executive functioning through the Frontal Assessment Battery (FAB), a traditional standardized paper-and-pencil test specific for EFs [46,47]. After that, the psychologist collected participants’ socio-demographic data (e.g., age, gender, education level) and technological expertise through an ad hoc questionnaire in which they had to evaluate their perceived level of familiarity and competence with several technologies: tablet, smartphone, computer, and the Internet. Specifically, the questionnaire involved a 5-point scale (from “never” to “every day”) in evaluating “how often, in the last year, did you use...” and a 5-point scale (from “nothing” to “much”) to investigate “how competent do you feel in using...”.

After the preliminary screening, all participants underwent an evaluation session with EXIT 360°. The psychologist started the administration by inviting participants to sit on a swivel chair and wear the mobile-powered headset. Before wearing the headset, participants received a general instruction of the task: “*You will now wear a headset. Inside this viewer, you will see some 360° rooms of a house. To visualize the whole environment, I ask you to turn on yourself; you are sitting on a swivel chair for this reason. Within these environments, you will be asked to perform some tasks.*”

After that, participants started an initial phase to familiarize themselves with the device and virtual environment and to control any adverse effects (e.g., dizziness, nausea). Participants were immersed in a neutral 360° living room, exploring the settings and finding specific objects. At the end of this preliminary phase, participants had to indicate the presence of any negative sides. If the subjects did not report side effects, they were immersed in another 360° living room. They started the real experimental session, hearing the following instruction: “*You are about to enter a house. Your goal is to get out of this house in the shortest time possible. To exit, you will have to complete a path and a series of tasks that you will encounter along your way. Are you ready to start?*”. All instructions were provided in a standardized way, as they have been previously recorded and inserted within the virtual environments. 

During their evaluation, the subjects were engaged in many domestic 360° environments explorable through the head’s movement as in real-life situations [48]. In these environments, participants had to perform seven everyday subtasks of increasing complexity that wanted to tap and evaluate different EFs (for a detailed description, see [32]). 

**Figure 1:**
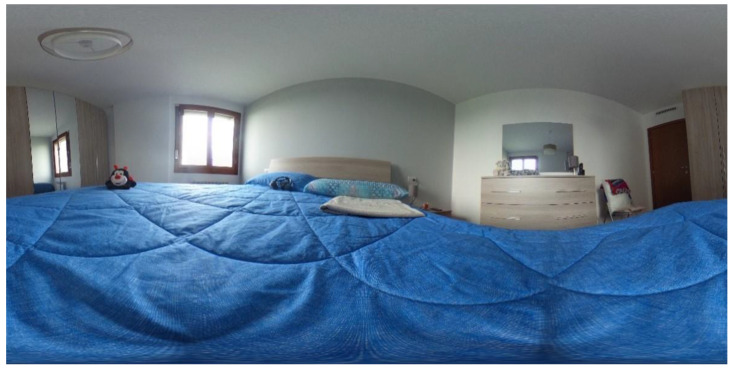
A representation of a 360° environment that participants see in the headset (here, the image is represented in anamorphic format).

Briefly, “Let’s Start” requires subjects to observe a map, choosing the path that allows them to reach the “finish” in the shortest possible time. In the second subtask, “Unlock the Door”, the participants have to open a door choosing between “key, telephone, and drill”. The task “Choose the Person” requires the participant to explore a living room and select a specific person according to a particular instruction. In task 4, “Turn on the Light”, the subjects are immersed in a dark room because “the power went out,” and they must choose the object that allows them to continue the journey. In the following task (“Where are the Objects?”), participants have to identify the piece of furniture on which four specific objects (i.e., telephone, lamp, teddy bear and blanket) are placed in a bedroom (Figure 1).

In task 6, subjects must complete a rebus. Finally, they must memorize a sequence of numbers in the last task, “Create the Sequence”, reporting them in reverse. 

**Figure 2:**
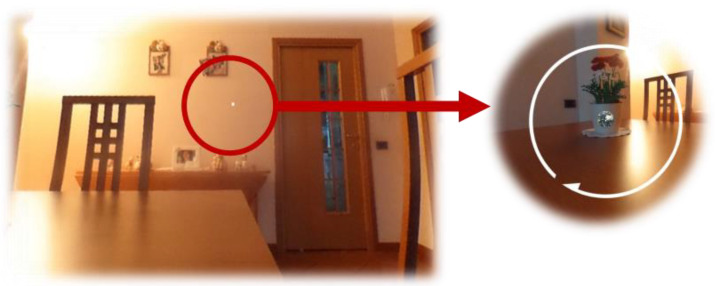
The representation of the small white dot that participants see in the headset. To respond, participants must move their heads and position the dot on the answer for a few seconds and the answer will be selected automatically.

EXIT 360° was designed to allow participants to respond to each task by choosing between three or more alternatives simply by moving their head and positioning a small white dot that they saw in the headset on the answer for a few seconds (Figure 2). Therefore, the answer will be automatically selected, and participants will not have to learn to use complex tools. 

Participants had to perform all seven subtasks, obtaining one point for a wrong answer or two for a correct one. Therefore, we evaluated the usability and user experience of the whole task. Overall, EXIT 360° allowed for the collection of Total Score (range 7–14) and Total Reaction Time (i.e., time in seconds registered from examiner’s instruction until the participant provided the last correct answer). 

After the EXIT 360° session, participants underwent an evaluation that assessed usability and user experience quality. Regarding usability assessment, we used the System Usability Scale (SUS), a short questionnaire of 10 items on a 5-point scale from “completely disagree” to “strongly agree”, often used to evaluate the overall usability of technological instruments [49,50,51]. Furthermore, SUS allowed for evaluation of the two main aspects that could affect the user experience: usability (easy to use the system) and learnability (easy to learn to use the system) [51]. Table 1 shows the questionnaire and scale used to evaluate the user experience. 

### 2.3. Statistical Analysis

Descriptive statistics included frequencies, percentages, and median and interquartile range (IQR) for categorical variables and mean and standard deviation (SD) for continuous measures. The normality of data distribution was assessed using the Kolmogorov–Smirnov test. A *t* test for independent sample (parametric or non-according to variables) and Chi-square were conducted to verify possible differences between pathological group and healthy controls in main demographic and clinical characteristics and technological expertise. Moreover, Pearson’s correlation was applied to compare the usability scores, user experience, and technological experience. At the same time, a *t* test for the independent sample was conducted to evaluate any significant differences between groups in the same variables. All statistical analyses were performed using Jamovi 1.6.7 software. A statistical threshold of *p* < 0.05 was considered statistically significant.

## 3. Results

### 3.1. Participants

Table 2 reports the demographic and clinical characteristics of the whole sample (N = 54), divided into two groups. PwPD (*n* = 27) was predominantly female (M:F = 11:16) with a mean age of 68.2 (SD = 9, range = 53–84) and age of education =13 (IQR = 5, range 5–18). HC was predominantly female (M:F = 11:16) with a mean age of 66.4 (SD = 10.5, range = 48–88) and age of education = 13 (IQR = 5, range 5–18). The comparison between the PwPD and HC showed the absence of significant differences in all main demographic and clinical characteristics. All participants included in the study showed no cognitive impairment (cutoff of normality = MoCA score ≥ 17.54).

### 3.2. Technological Expertise

**Figure 3:**
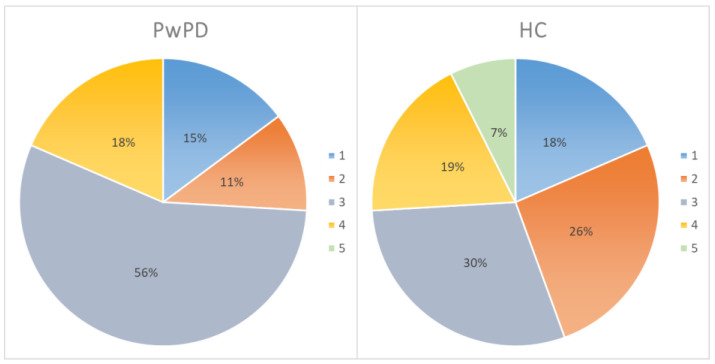
Percentages relating to familiarity with the technologies for each group. PD, Parkinson’s disease; HC, healthy control. 1 = never; 2 = once a month or more rarely; 3 = once a week; 4 = every 2/3 days; 5 = every day.

The ad hoc five-point scale for evaluating the perceived level of familiarity with technologies showed similar results between patients and healthy controls, with a mean score of 3.15 ± 0.89 and 3.14 ± 1.12 (i.e., participants used the technology about once a week). Figure 3 shows the percentages relating to familiarity with the technologies for each group.

Moreover, the mean score of the ad hoc five-point competence questionnaire was 2.68 ± 1.01 for PwPD, indicating a score near to “little”. HC obtained a mean score of 3.04 ± 0.98 (i.e., neither enough nor little). 

**Figure 4:**
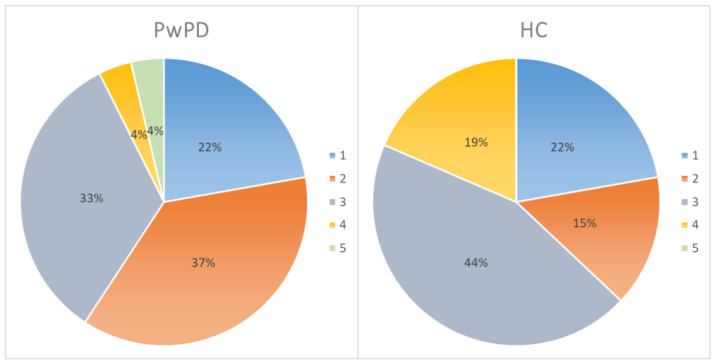
Percentages relating to the self-reported competence in using several technologies for each group. PD, Parkinson’s disease; HC, healthy control.

Figure 4 shows the percentages relating to the self-reported competence in using several technologies for each group. Overall, only 7.4% of PwPD and 18.5% of HC showed a good (≥4—enough or much) competence with technology.

Analyzing the possible differences between groups, data showed the absence of significant differences in levels of competence (*t* test (52) = −1.377; *p* = 0.174) and familiarity (*t* test (52) = 0.045; *p* = 0.964) with technologies.

### 3.3. Neuropsychological Evaluation

Preliminary analyses have shown that EXIT 360° could be considered as an effective tool in discriminating between PwPD and HC. Table 3 showed significant differences between the two groups in Total EXIT score (*t* test (52) = −4.95; *p* < 0.001) and Total Reaction time (*t* test (52) = 7.12; *p* < 0.001). Specifically, HC obtained a higher total score (mean = 12.3 ± 1.07) and completed the test in less time (mean = 457.3 ± 73.60). Furthermore, a significant difference also appeared in the FAB score (*p* = 0.006), showing that HC achieved a higher performance (17.46 ± 1.003).

### 3.4. EXIT 360°: Usability

**Figure 5:**
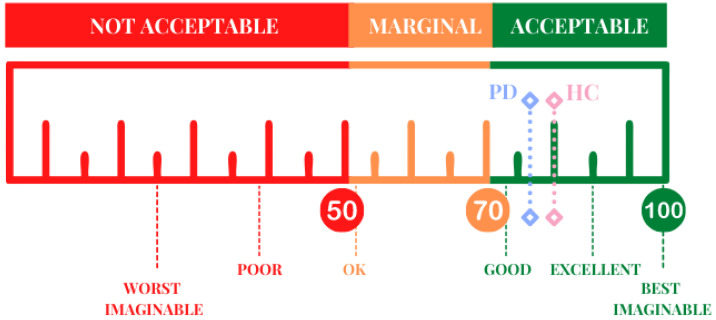
A graphic representation of the SUS score. PD, Parkinson’s disease; HC, healthy control.

Figure 5 shows the mean value of the usability provided by both groups at the SUS. The comparison between the PwPD and HC showed the absence of significant difference in usability score (*t* test (52) = −1.09; *p* = 0.279). PwPD provided a mean score of 76.94 ± 9.18, while HC showed a mean score of 80 ± 11.22. Both scores indicate a satisfactory level of usability, according to the scale’s score acceptability ranges (cut off = 68) and adjective ratings (included between “good” and “excellent”).

Specifically, according to the cut-off score (cut-off = 68), more than 74% of PwPD and 92% of HC showed scores above the cut-off. In addition, according to the adjective rating, 29.6% of subjects evaluated EXIT 360° as “OK”, 59.3% as “good”, 7.4% as “excellent”, and 3.7% as “best imaginable” [51,58]. As regards HC, 3.7% of subjects evaluated EXIT 360° as “OK”, 55.6 as “good”, 18.5% as “excellent”, and 18.5% “best imaginable”, with only one participant that showed a low score. Finally, participants provided good and promising scores for two main aspects affecting the user experience. The comparison between groups showed the absence of significant differences in usability (*t* test (52) = −1.96; *p* = 0.055) and learnability (*t* test (52) = 1.89; *p* = 0.064). Specifically, PwPD showed a mean score of 2.98 ± 0.47 for usability (vs 3.24 ± 0.50) and 3.37 ± 0.63 for learnability (vs. 3.06 ± 0.59). Only 11.1% of patients and 3.7% of controls showed low scores (<2.5) at usability, while only 3.7% for each group in learnability.

### 3.5. EXIT 360°: User Experience

The first item of the Flow Short Scale showed a high score in the perceived level of skill in performing EXIT 360° both for PwPD and HC (median = 5, IQR = 4–5), without significant difference between groups (U test (52) = 363; *p* = 0.978). In addition, the other two items allowed for evaluation of the level of challenge of EXIT 360°, also based on their own abilities, such as balance/appropriate (median = 3; IQR = 3) for both groups, without significant difference (U test (52) = 326; *p* = 0.191).

Table 4 showed that the subscale enjoyment of the IMI obtained high scores (≥4) in all items without significant differences between the two groups.

**Figure 6:**
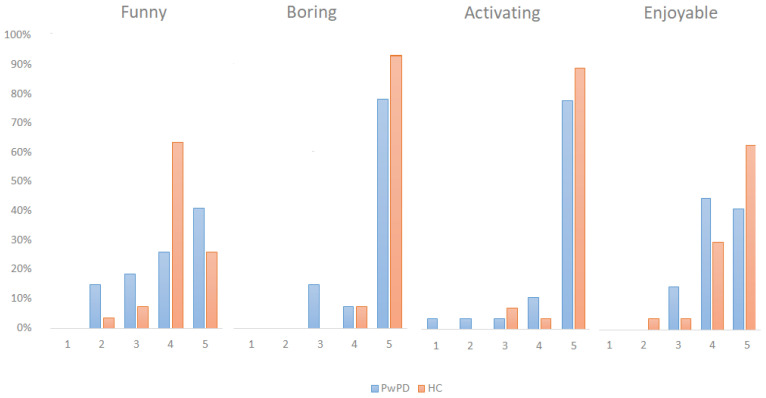
Graphic representation of the Intrinsic Motivation Inventory domains, comparing patients and controls.

Specifically, Figure 6 showed the percentage relating to all four items of subscale enjoyment of the IMI, comparing two groups. The figure emerges that both PwPD and HC considered EXIT 360° as activating, funny and enjoyable. No participant evaluated EXIT 360° as boring. 

Table 5 showed good scores in all ICT—SOPI dimensions. The comparison between the PwPD and HC showed a significant difference only in the domain “engagement” (*t* test (52) = −3.44; *p* < 0.05). 

As regards the domain “negative effects”, only a few participants (three PwPD and three HC) reported the presence of minor adverse effects (score < 3), such as vertigo or nausea. 

**Figure 7:**
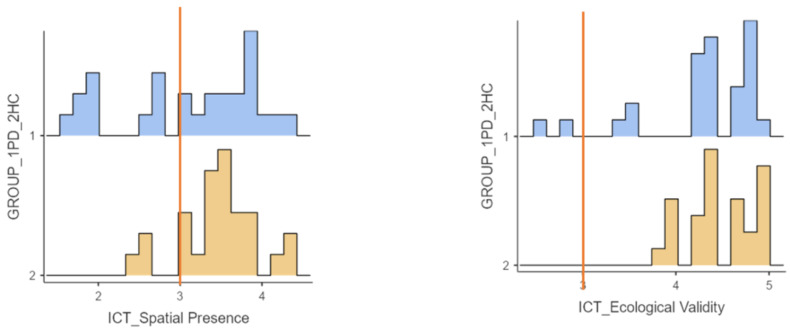
Graphic representation of two ICT—SOPI dimensions. The orange lines indicate a neutral score.

Figure 7 showed participants’ good and promising scores at domains “spatial presence” and “ecological validity”, divided according to groups. First, most of the participants (70.4 of PwPD and 88.9 of HC) showed good scores in terms of spatial presence (≥3—e.g., (“I felt I could interact with the environment shown”). In addition, all healthy control subjects and 92.6% of patients supported that EXIT 360° had good ecological validity (“I had the feeling that the environment shown was part of the real world”), with most of the participants (respectively, 96.3% and 85.2%) that provided high scores (≥4).

Finally, despite the two groups that obtained a significant difference in the domain “engagement”, most of the participants indicated a good level of engagement while performing EXIT 360° (≥3—e.g., “I would have liked the experience to continue”), with only six patients and one control that showed low scores.

**Figure 8:**
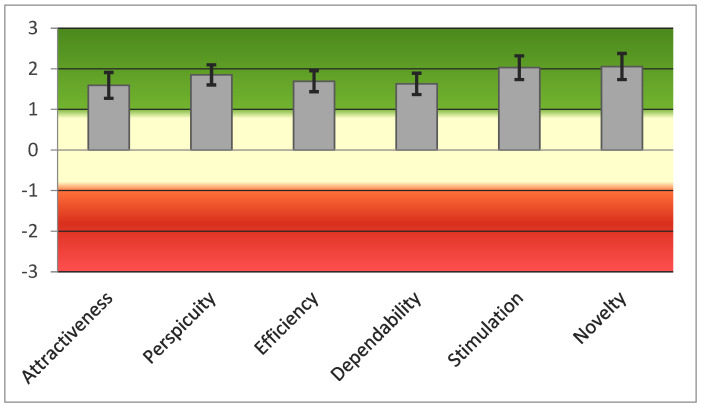
Graphic representation of scores of the six UEQ scales.

The UEQ questionnaire showed positive evaluation (>0.8) in all 26 items in both groups. Figure 8 showed good scores obtained by PwPD in all UEQ scales according to the questionnaire’s score ranges (range between −3, horribly bad, and +3, extremely good).

Table 6 shows in detail the high mean scores of all scales (regarding PwPD) with their respective good values of internal consistency (Alpha-coefficient > 0.7) [59].

Table 7 shows good scores in all UEQ scales and two main dimensions (pragmatic and hedonic quality) of patients and healthy subjects, including the comparison between two groups. Specifically, UEQ’s scales can be grouped into pragmatic quality (perspicuity, efficiency, dependability) and hedonic quality (non-task-related quality aspects—stimulation and originality).

**Figure 9:**
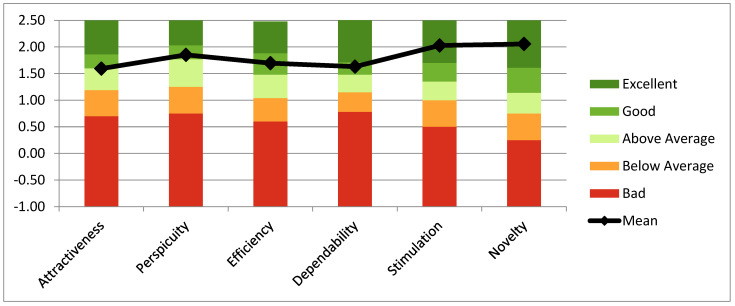
Comparison between means of each UEQ scale and values from a benchmark dataset.

Finally, the means of each UEQ scale of PwPD were compared to existing values from a benchmark dataset (containing data from 20,190 persons from 452 studies) [60]. Results showed that the scale’s stimulation and novelty obtained excellent evaluation, belonging to the range of the 10% best results (Figure 9). Moreover, the scales for Attractiveness, Perspicuity, Efficacy and Dependability obtained a good evaluation, that is, “10% of results better, 75% of results worse.”

### 3.6. Correlation

Overall, Pearson’s correlation showed the absence of significant linear correlation between the SUS total score and education (r = 0.078; *p* = 0.576), but not for age (r = −0.401; *p* < 0.05). Moreover, Pearson’s correlation has underlined the absence of significant correlation between SUS total score and technological expertise measured by the ad hoc questionnaire of competence, both for patients (r = 0.340; *p* = 0.082) and controls (r = 0.244; *p* = 0.221). As regards the relationship between usability and user experience, patients showed no linear correlation between SUS total score and three ICT—SOPI domains, spatial presence (r = 0.293; *p* = 0.138), engagement (r = 0.361; *p* = 0.064), and ecological validity (r = 0.282; *p* = 0.154). HC obtained similar results, except for ecological validity that appeared significantly correlated with usability score (r = 0.422; *p* = 0.028). Moreover, data showed the presence of a significant and negative correlation between the SUS total score and the ICT—SOPI domain negative effect only in PwPD (r = −0.325; *p* < 0.05). Finally, the statistical analysis showed the absence of significant correlations between usability and the three dimensions of UEQ: attractiveness (r = 0.168; *p* = 0.224), pragmatic quality (r = 0.196; *p* = 0.157) and hedonic quality (r = 0.250; *p* = 0.069).

## 4. Discussion

Executive dysfunction represents a typical non-motor symptom in PD, impacting everyday functioning and quality of life from the early stages of the disease course [5,7,13,14,15]. Several studies have shown the feasibility, acceptability, and efficacy of VR-based tools for an early and ecologically valid evaluation of ED in many neurologic pathologies [4,5,6,25,26]. In this framework, we have developed EXIT 360° that fits perfectly into the ongoing transformation of traditional neuropsychological assessment [2,23,24]. EXIT 360° aimed to be an original 360°-based instrument for a multicomponent, ecologically valid evaluation of executive functioning in Parkinson’s disease [32]. EXIT 360° have overcome the first validation steps to become a valuable and standardized instrument for assessing EFs, showing excellent convergent validity and promising usability and user experience results in a healthy sample [33]. These results appear interesting because many studies have demonstrated the need to consider the evaluation of “usability” and “user experience” as crucial elements in developing technological tools [34,35,36,37]. Their evaluation allows for understanding any technical (e.g., technological expertise) or clinical (e.g., side effects or motivation) elements that could affect the users’ performance and significantly decrease the test results’ validity [36,38,39,40]. Therefore, we have focused on evaluating usability and user experience in a sample of patients with PD.

Our work involved twenty-seven patients with PD, matched with twenty-seven healthy controls subjects. All subjects involved in the study met the inclusion criteria and successfully carried out EXIT 360° without relevant adverse effects, as demonstrated by previous studies with 360°-based instruments [25]. At the baseline, the whole sample showed low technological expertise regarding the perceived level of familiarity (i.e., participants used the technology about once a week) and technology competence. Only 7.4% of PwPD and 18.5% of HC showed good competence with technology. Overall, despite the low level of familiarity and competence with the technologies, all subjects were able to complete the test successfully. Therefore, EXIT 360° appeared as a promising tool that clinicians could use even with patients without a great technological experience.

Regarding usability evaluation, data showed a good usability score, evaluated by SUS, for both groups without significant difference. PwPD provided a mean score of 76.94 ± 9.18, while HC showed a mean score of 80 ± 11.22. Both scores indicate a satisfactory level of usability, according to the scale’s score acceptability ranges (cut off = 68) and adjective ratings (included between “good” and “excellent”) [49]. Specifically, according to the adjective rating, 29.6% of patients evaluated EXIT 360° as “OK”, 59.3% as “good”, 7.4% as “excellent”, and 3.7% as “best imaginable” [58]. Moreover, all participants provided good and interesting scores for the variables of usability and learnability, indicating that EXIT 360° can be considered as an easy-to-use technological tool and an easy-to-learn instrument [51] for the patients. All these promising usability results allow us to conclude that EXIT 360° showed high effectiveness (i.e., possibility for the users to achieve goals), efficiency (i.e., users’ efforts to reach the aim), and satisfaction (i.e., users’ thoughts about their interaction with the system) [35,38,50]. Thus, it is possible to support that any subject’s low performance does not depend on technological problems. The usability result was not influenced by education level and technological expertise measured by the ad hoc questionnaires of competence. Only the age variable showed a negative correlation with usability score; however, older people (both patients and controls) were able to complete the evaluation with some instructions. Overall, according to these results, no adaptation of our system would be necessary. These findings appeared interesting and promising compared to the only previous usability study on PD and VR-based instruments [36], in which data showed that healthy participants gave good usability for the VR-based tool, while the PwPD showed that there needs to be more than an improvement to the instrument to be considered usable.

In addition to the good usability level, EXIT 360° showed promising results in terms of user experience in both groups. First, patients with PD showed high scores in the perceived level of skill in performing EXIT 360° and evaluated the EXIT 360° subtasks as balanced/appropriate concerning their abilities. Second, they supported a positive general impression of the EXIT 360°, considering it attractive (e.g., pleasant, pleasing, friendly, and enjoyable), activating, funny and not boring. Moreover, EXIT 360° demonstrated a good pragmatic quality as it appeared: (1) efficient, fast, practical, and organized (efficiency); (2) understandable, easy to learn, and straightforward (perspicuity); and (3) predictable, supportive, and secure (dependability). The two groups obtained similar scores in these variables, except for dependability, in which patients obtained a lower score. However, this domain was influenced by the item “meets expectations” because patients referred to having negative expectations due to traditional long and complex evaluation, and they claimed to be pleasantly surprised. In addition, EXIT 360° showed excellent hedonic quality in terms of stimulation (valuable, exciting, interesting, and motivating) and novelty (creative, innovative, inventive). Interestingly, results showed that the scales stimulation and novelty evaluated better than existing values from benchmark data [60]. Finally, all participants considered EXIT 360° as an engaging and challenging tool with good spatial presence (“I felt I could interact with the environment shown”) and as excellent (“I had the feeling that the environment shown was part of the real world”), with most of the participants (96.3% controls and 85.2% patients) providing high scores. Moreover, despite that the two groups obtained a significant difference in the domain “engagement”, most of the participants indicated a good level of engagement while performing EXIT 360° (e.g., “I would have liked the experience to continue”), with only six patients and one control showing low scores. However, the six patients claimed that “the evaluation had a correct duration but that they would have had no problems continuing”. As regards adverse effects, only three PwPD and three HC reported the presence of irrelevant adverse effects, such as vertigo or nausea. These results appear promising considering previous literature that supports the importance of the sense of presence/realism, engagement, enjoyment, and side effects in digital content development [39,61].

Overall, the present results appear promising in terms of usability and user experience of EXIT 360° in patients with PD, in line with results obtained in the previous study on healthy controls. Briefly, EXIT 360° allowed for an ecologically valid evaluation without relevant sicknesses (e.g., dizziness, headache, and nausea) that lead to unpleasant experiences for the patients, impacting their performance and significantly decreasing the test results’ validity [23]. Moreover, our results showed that technological expertise does not affect the EXIT 360° performance [40]. In addition, the high levels of enjoyment, engagement, and attractiveness of EXIT 360° allowed for increasing users’ motivation and participation and decreasing anxiety typical of neuropsychological evaluation. These results supported the innovative higher-level concept of interaction experience, proposed by Sauer and colleagues, in which usability and user experience can be considered key elements to provide more benefits in using technological tools [35].

### Limitations and Future Perspectives

The present work has some limitations. First, the head-mounted display used was entry level: the 360° mobile-powered devices currently available on the market have higher quality (e.g., Oculus Quest), which would allow for improving the quality of 360° images, providing a better and more realistic experience. Second, possible misdiagnosis with syndromes showing high resemblance with PD (e.g., Parkinsonism variant of Progressive Supranuclear Palsy (PSP-P)) could have an impact on enrollment and results. Possible misdiagnosis is an important concern for experienced movement disorder specialists, particularly in the early stage of the disease in which several clinical manifestations could overlap [62,63]. To minimize possible misdiagnosis in this study, the patients’ enrollment was made by an experienced neurologist of the Parkinson Center of the Fondazione Don Carlo Gnocchi, according to clinically established or probable PD MDS criteria. Finally, to date, EXIT 360° can be considered as a promising prototype that needs other validation steps to become a valid and standardized instrument for assessing EFs. For this reason, it will be necessary to deepen its effectiveness in discriminating between healthy control subjects and patients with executive dysfunctions. 

## 5. Conclusions

The present study results are promising and interesting in terms of the usability and user experience of EXIT 360° in patients with PD. Overall, patients provided a positive global impression of the instrument, evaluating it as usable, easy-to-learn, understandable, enjoyable, attractive, and friendly. Moreover, EXIT 360° is an efficient, fast, and organized tool, with excellent hedonic quality regarding stimulation (exciting and interesting) and novelty. Finally, EXIT 360° also appeared to be an engaging and challenging tool with good spatial presence, excellent ecological validity, and irrelevant adverse effects. Technological expertise did not influence the encouraging results. Further studies will have to be conducted to deepen the efficacy of EXIT 360° in discriminating between healthy control subjects and patients with executive dysfunctions.

## Figures and Tables

**Table 1:** Questionnaire and scale to evaluate the user experience.

Scale	Aim	Characteristics
*User Experience Questionnaire (UEQ)* [52,53,54]	1. attractiveness (overall impression of the product) 2. perspicuity: easily to learn how to use the product 3. efficiency (user’s effort to solve tasks) 4. dependability (feeling of control of the interaction) 5. stimulation (motivation to use the product) 6. novelty: (innovation and creation of product)	a 26 item-scale (semantic differential scale: each item consists of two opposite adjectives, e.g., boring vs. exciting) that allows for calculation of the six different domains
*ICT—Sense of Presence Inventory (ICT—SOPI)* [55]	1. spatial, physical presence: the feeling of being in a physical space in the virtual environment and having control over it 2. engagement: the tendency to feel psychologically and pleasantly involved in the virtual environment 3. ecological validity: the tendency to perceive the virtual environment as real 4. negative effects: adverse psychological reactions	44 item-scale 5-point scale from 1: “strongly disagree” to 5 “strongly agree.” ICT—SOPI is divided into thoughts and feelings after experiencing the environment (Part A) or while the user was experiencing the environment (Part B). Items are divided into four dimensions, generated by calculating the mean of all items contributing to each factor.
*Flow Short Scale (three items)* [56]	perceived level of: —abilities in coping with the task —challenges —challenge-skill balance	5-point scale: from low to high
*Intrinsic Motivation Inventory (subscale enjoyment—four items)* [57]	participants’ appreciation of the proposed activity (i.e., boring, pleasant, fun and activating)	5-point scale: from low to high The item boring scores were reversed to align with the remaining items; therefore, in the whole scale, a low value in the items reflects a negative perception of the experience with EXIT 360°.

**Table 2:** Demographic and clinical characteristics of the whole sample.

	PwPD N = 27	HC N = 27	Group Comparison (*p*-Value)
**Age** (years, mean (SD))	68.2 (9)	66.4 (10.5)	0.507
**Sex** (M: F)	11:16	11:16	1.000
**Age of education** (years, median (IRQ))	13 (5)	13 (5)	0.740
**MoCA_raw score** (mean (SD))	25.4 (3.12)	26.3 (2.25)	0.235
**MoCA_adjusted score** (mean (SD))	25.3 (2.25)	26.0 (2.53)	0.246

M, male; F, female; SD, standard deviation; IQR, interquartile range; *n*, number; MoCA, Montreal Cognitive Assessment; PwPD, patients with Parkinson’s disease; HC, healthy controls.

**Table 3:** Comparison of scores at EXIT 360°.

	PwPD Mean ± SD	HC Mean ± SD	Group Comparison (*p*-Value)
**Total EXIT Score**	10.5 ± 1.58	12.3 ± 1.07	**<0.001**
**Total Reaction Time**	716.4 ± 174.19	457.3 ± 73.60	**<0.001**
**FAB**	15.94 ± 2.33	17.46 ± 1.003	**0.006**

SD, standard deviation; PwPD, patients with Parkinson’s disease; HC, healthy controls; FAB = Frontal Assessment Battery (in bold, statistically significant value).

**Table 4:** Comparison of scores at the subscale enjoyment of IMI.

	PwPD Median (IRQ)	HC Median (IRQ)	Group Comparison (*p*-Value)
**Boring**	5 (5)	5 (5)	0.107
**Enjoyable**	4 (4–5)	5 (4–5)	0.113
**Activating**	5 (5)	5 (5)	0.28
**Funny**	4 (3.5)	4 (4–4.5)	0.81

IQR, interquartile range; *n*, number; PwPD, patients with Parkinson’s disease; HC, healthy controls.

**Table 5:** Comparison of scores in ICT—SOPI dimensions.

	PwPD Mean ± SD	HC Mean ± SD	Group Comparison (*p*-Value)
**Spatial Presence**	3.11 ± 0.83	3.47 ± 0.48	0.054
**Engagement**	3.43 ± 0.54	3.9 ± 0.47	**0.001**
**Ecological Validity**	4.29 ± 0.61	4.49 ± 0.37	0.149
**Negative Effects**	1.29 ± 0.42	1.2 ± 0.26	0.361

SD, standard deviation; PwPD, patients with Parkinson’s disease; HC, healthy controls (in bold, statistically significant value).

**Table 6:** Scores of the six UEQ scales. SD, standard deviation.

	Mean	SD	Confidence Interval		Alpha- Coefficient
**Attractiveness**	1.593	0.846	1.273	1.912	0.89
**Perspicuity**	1.852	0.655	1.605	2.099	0.81
**Efficiency**	1.694	0.681	1.438	1.951	0.72
**Dependability**	1.630	0.695	1.368	1.892	0.78
**Stimulation**	2.028	0.776	1.735	2.321	0.79
**Novelty**	2.056	0.853	1.734	2.377	0.93

**Table 7:** Comparison of scores in UEQ scales and dimensions.

	PwPD Mean (SD)	HC Mean (SD)	Group Comparison (*p*-Value)
**Attractiveness**	1.59 (0.85)	1.81 (1.13)	0.430
**Perspicuity**	1.85 (0.66)	2.01 (0.75)	0.416
**Efficiency**	1.69 (0.68)	1.73 (0.84)	0.859
**Dependability**	1.63 (0.70)	2.14 (0.86)	**0.020**
**Stimulation**	2.03 (0.78)	2.08 (0.98)	0.818
**Novelty**	2.06 (0.85)	2.46 (0.68)	0.058
**Pragmatic Quality**	1.73 (0.59)	1.96 (0.74)	0.204
**Hedonic Quality**	2.04 (0.72)	2.27 (0.82)	0.275

SD, standard deviation; PwPD, patients with Parkinson’s disease; HC, healthy controls (in bold, statistically significant value).

## Data Availability

Data can be obtained upon reasonable request to the corresponding author.

## Abbreviations

ED	Executive Dysfunctions
EFs	Executive Functions
EXIT 360°	EXecutive-functions Innovative Tool 360°
F	Female
FAB	Frontal Assessment Battery
HC	Healthy Control
ICT—SOPI	ICT—Sense of Presence Inventory
IMI	Intrinsic Motivation Inventory
IQR	Interquartile Range
M	Male
MDS	Movement Disorder Society
MoCA test	Montreal Cognitive Assessment Test
N	Number
PD	Parkinson’s Disease
PIT	Picture Interpretation Test
PwPD	Patients with Parkinson’s Disease
SD	Standard Deviation
SUS	System Usability Scale
UEQ	User Experience Questionnaire
VMET	Virtual Multiple Errand Test
VR	Virtual Reality
